# Social and clinical factors associated with non-adherence to radiotherapy with curative intent in a cancer centre in Peru

**DOI:** 10.3332/ecancer.2026.2081

**Published:** 2026-02-24

**Authors:** Robles Díaz, José Fernando, Flores Romaní, Lucero Silvana

**Affiliations:** Regional Institute of Neoplastic Diseases of the Center, Concepción, Junín 12125, Perú

**Keywords:** Cancer care, adherence to radiotherapy, indigenous populations, health inequalities, curative intent, progression-free survival, Peru

## Abstract

**Background:**

Adherence to radiotherapy with curative intent is essential to achieve optimal tumour control and improve survival. In low- and middle-income countries, particularly in rural and multicultural settings, structural and sociocultural barriers can significantly compromise treatment completion.

**Objective:**

To identify the social and clinical factors associated with non-adherence to radiotherapy with curative intent and its impact on progression-free survival in patients treated at a Peruvian cancer centre.

**Methods:**

A retrospective observational study was conducted that included 254 patients treated with radiotherapy with curative intent between 2020 and 2021. Data were obtained through structured review of medical records and validated surveys. Multivariate logistic regression and survival analysis using Kaplan–Meier curves were applied.

**Results:**

Non-adherence was significantly associated with belonging to an indigenous ethnic group (odds ratio (OR): 10.45; 95% confidence interval (CI): 2.34–46.67), lack of access to paved roads (OR: 2.19; 95% CI: 1.10–4.36) and incomplete secondary education (OR: 2.31; 95% CI: 1.33–4.01). These factors were also associated with reduced progression-free survival.

**Conclusions:**

Structural and sociocultural determinants significantly influence adherence to radiotherapy in underserved populations. Tailored interventions—such as transport support, intercultural health education and the implementation of hypofractionated protocols—are urgently needed to improve equity in cancer care.

## Introduction

Adherence to radiotherapy regimens with curative intent is a determining factor in the efficacy of cancer treatment. Globally, treatment interruptions have been shown to negatively affect tumour control and survival, particularly in neoplasms such as cervical, breast and head and neck cáncer [1–3]. The fractionated nature of radiotherapy makes adherence even more critical, as delays or dropouts compromise the dose-time relationship and reduce the likelihood of local control.

Previous studies in Asia and Europe have reported non-adherence rates ranging from 15% to 40% depending on the social and economic context [4, 5]. In Latin America, research in Colombia and India shows that the most common barriers are related to poverty, road inaccessibility and membership in indigenous communities [6–8]. However, there is little systematic evidence on this problem in Peru, a country with marked geographical and cultural diversity, where the population of the highlands and jungle faces particular obstacles in accessing complex treatments such as radiotherapy [9,10].

In this scenario, it is essential to analyse the clinical and social determinants that limit adherence. This study seeks to identify these factors in patients treated with curative intent at a cancer centre in Peru and to evaluate their impact on survival, in order to generate evidence applicable to other middle-income countries with similar realities.

## Materials and methods

The study protocol was previously approved by the Institutional Ethics Committee of the Radiotherapy Centre.

The study was observational, analytical and retrospective. The population consisted of 412 cases with curative intent that met the selection criteria. Inclusion criteria: Pathological anatomy with a diagnosis of malignant neoplasm, patients received radiation scheduling consultations between 2020 and 2021 and patients had to belong to the institution's jurisdiction. Exclusion criteria: Age under 18 years, metastasis from unknown primary, no correct indication for radiation, pregnant women, suspension during external radiotherapy due to neoplasia progression, abandonment of external radiotherapy due to non-medical death, having received external radiotherapy on an outpatient basis in less than 90% of sessions, and suspension of external radiotherapy due to a confirmed case of COVID-19.

The sample size was calculated with a confidence level of 0.99% and a margin of error of 5%. Cases were selected probabilistically, using simple random sampling. For the study, cases had to be identified in order to request their informed consent to participate in the study. Priority was given to obtaining consent from the patient if they were alive or from a first-degree relative if the patient had died. After informed consent was obtained, a survey validated by experts was administered to collect information not available in the medical record, such as: indigenous population, productive activity, secondary education completed, road access and reason for interruption.

The following variables were defined:

- Sex (male/female). Obtained from the medical record.
- Age (years). Obtained from medical records.
- Indigenous affiliation (yes/no). Obtained from the survey.
- Productive activity (agriculture/commerce/manufacturing/construction, textiles/unspecified). Obtained from the survey.
- Residence in provincial capital (yes/no). Obtained from geolocation.
- Access to paved roads (yes/no). Obtained from geolocation.
- Completed secondary education (yes/no). Obtained from the survey.
- Tumour site (cervix/head and neck/skin/prostate/other). Obtained from medical history.
- Clinical stage (I/II/III/IV/progression). Obtained from medical history.
- Concurrent chemotherapy (yes/no). Obtained from medical history.
- Interruption (yes/no): Interruption was considered if at least one day of external radiotherapy had been missed. If the patient had had an interruption due to linear accelerator failures or holidays, this was obtained from the medical history record at; otherwise, it was obtained during the survey. Obtained from medical history.
- Adherent (yes/no): The case was considered non-adherent when it met at least one of the following criteria: Start date postponed for non-medical reasons, never started external radiotherapy for non-medical reasons, suspension of external radiotherapy within 2 days for any reason, less than 90.0% of the indicated external radiotherapy sessions completed for non-medical reasons or abandonment of external radiotherapy. Obtained from the medical record.
- Progression-free survival: months from the start of radiotherapy to progression. Obtained from medical records.
- Overall survival: months from the start of radiotherapy to death. Obtained from medical records.

All data were analysed using the SPSS statistical programme (version 28.0, Chicago, IL, USA). Simple and multivariate logistic regression was performed between the factors and adherence. To compare the survival means between the factors, Student's *t*-test and analysis of variance (ANOVA) were performed for dichotomous and polytomous variables, respectively. Survival analysis was constructed from Kaplan–Meier curves.

It was conducted following the guidelines of the STROBE observational study.

## Results

The sample of cases that met the evaluation criteria was 254. The general characteristics are shown in Table 1. Of note are the indigenous origin of 7.9%, residence in the provincial capital of 70.1%, access to paved roads of 54.7%, completion of secondary education of 61.8% and adherence of 56.3%.

Table 2 shows the risk analysis of the factors for non-adherence. In the simple model, the following factors were significant: indigenous ethnicity, lack of access to paved roads and lack of complete secondary education. However, when subjected to the multivariate model, indigenous ethnicity was significant (odds ratio (OR): 10.45 confidence interval (CI): 2.34–46.67), lack of access to paved roads (OR: 2.19 CI: 1.10–4.36) and lack of complete secondary education (OR: 2.31 CI: 1.33–4.01).

Table 3 presents the assessment of progression-free survival with statistically significant factors: indigenous membership (6.50 months) versus non-indigenous membership (31.04 months), agricultural production activity (19.91 months) versus trade (29.99 months) versus manufacturing (38.79 months) versus construction (44.47 months) versus textiles (19.64 months), no access to paved roads (18.49 months) versus access to paved roads (37.90 months), no secondary education (26.240 months) versus secondary education (33.76 months), tumour site in stomach–oesophagus (14.25 months) versus breast (43.78 months) versus cervix (29.22 months) versus head–neck (23.30 months), clinical stage I (41.44 months) versus II (44.94 months) versus III (25.17 months) versus IV (15.69 months) and progression (8.56 months), without adherence (24.49 months) versus with adherence (32.70 months), without interruption of external radiotherapy (34.70 months) versus personal reasons (22.310 months) versus logistical reasons (27.44 months). Likewise, overall survival is presented. When comparing the means, statistically significant results were found with a higher number of months for: non-indigenous membership, productive activity (agriculture and trade), residence in the provincial capital, paved road access, having breast cancer followed by skin cancer, having clinical stage I–II, with adherence, without interruption of external radiotherapy or where the interruption was due to holidays. Overall survival, when comparing the means, was statistically significant in favour of a higher number of months for: paved road access, having prostate cancer and adherence (Table 3).

**Figure 1.**
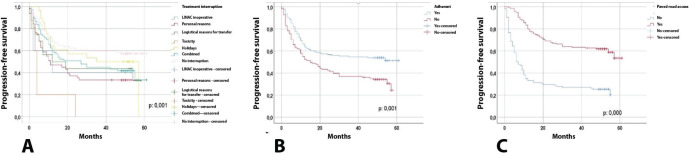
Progression-free survival. The values were significant with a p < 0.05.

**Figure 2.**
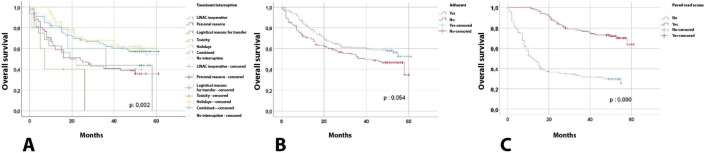
Overall survival. The values were significant with a p < 0.05.

Table 4 and Figure 1 present the comparison of progression-free survival, finding that the following variables were statistically significant: No interruption (39.69 months) versus interruption (19.43 months), no adherence (26.58 months) versus adherence (37.16 months) and no paved road access (19.19 months) versus paved road access (43.04 months). With regard to overall survival, the following variables were statistically significant: no interruption (43.12 months) versus interruption (32.22 months), no road access ( ) versus paved road access (50.76 months) (Figure 2).

## Discussion

This study is one of the first in Latin America to specifically analyse the social and clinical factors associated with non-adherence to radiotherapy with curative intent, and its impact on progression-free and overall survival. Our findings show that indigenous ethnicity, the absence of paved roads and lack of complete secondary education were the main factors associated with non-adherence. These structural and sociocultural determinants were related to lower progression-free and overall survival. Therapeutic adherence, in contrast, emerged as a key protective factor in survival, underscoring its clinical relevance.

The finding that two-thirds of patients discontinued their treatment reflects a worrying reality. This figure is comparable to that reported in European centres, where the discontinuation rate can reach 70%, although in very different contexts. In our study, the factors most strongly associated with non-adherence were belonging to indigenous peoples, the absence of paved roads and incomplete secondary education. These variables reflect not only the patient's geographical location but also their social, economic and cultural vulnerability.

Unlike in developed countries, where non-adherence is associated with characteristics such as advanced age or female gender [3], in our context, structural determinants predominated. The fact that indigenous membership has such a high OR (OR: 10.45) indicates a multifactorial barrier, possibly related to the cultural perception of cancer, access to medical information, language and interaction with the health system [5].

Road inaccessibility, even in areas considered provincial capitals, represents a critical physical barrier. It has been documented that living more than 50 km from the radiotherapy centre increases the risk of non-adherence [2], but in the present study, this situation is aggravated by the absence of paved roads, which limits daily or continuous transport, especially in patients with low economic resources and without institutional support [7, 11].

Likewise, lack of a complete secondary education may limit understanding of treatment, perception of cancer risk and the ability to plan family or work life in order to comply with the therapeutic sequence [8]. This variable also showed a strong association with non-adherence (OR: 2.31) and worse clinical outcomes, reinforcing the need for targeted and culturally adapted educational strategies [5].

When comparing our results with research in India and Colombia, similar patterns of inequality are evident in rural areas and among indigenous peoples [4–6]. In these contexts, solutions require more than clinical measures: a structural transformation of cancer care is needed, including institutional support, transport, accommodation and intercultural communication [9, 10, 12].

Therapeutic adherence was not only a primary outcome but also a key protective factor for survival, as shown by multivariate analyses and Kaplan–Meier curves. Adherent patients had significantly higher progression-free and overall survival than non-adherent patients, which is consistent with previous studies in breast and cervical cancer treated with radiotherapy [1, 13].

In terms of treatment interruptions, those motivated by personal or logistical reasons had the worst impact on survival, while technical interruptions (such as holidays or linear accelerator failures) had a less adverse effect. This reinforces the hypothesis that non-medical external barriers (such as transport, misinformation or lack of support) are the most detrimental in these types of vulnerable populations [2, 3].

One possible solution identified in other contexts and applicable here is the implementation of hypofractionated regimens, especially in tumours such as breast, cervix and rectum [1, 13, 14]. These regimens, already available at our centre, could reduce the total duration of treatment, facilitating compliance and reducing dropout.

Policy implications should be aimed at reinforcing the idea that structural and sociocultural factors have a greater impact than individual clinical characteristics in vulnerable contexts, underscoring the need for specific and sustainable institutional interventions. The implementation of hypofractionated regimens can reduce treatment duration and facilitate compliance. Likewise, free transport and accommodation programmes, staff training in intercultural communication and the participation of indigenous community promoters are feasible strategies with high potential for impact. These proposals, summarised in Table 5, respond to the need to address health inequalities from a comprehensive perspective.

### Limitations

This study has some limitations that should be considered when interpreting its results. Likewise, the retrospective component limits the possibility of establishing causal relationships between the factors evaluated and adherence or clinical outcomes. However, the linear accelerator management system records are available. The operational definition of adherence included a practical cut-off point of two days of suspension, in the absence of a standardised consensus on the number of days of interruption that clinically affects the efficacy of treatment in each type of tumour. This methodological limitation may have influenced the classification of some cases as non-adherent and, consequently, the survival analysis. In addition, other relevant variables such as nutritional status, comorbidity, income level and degree of family support, which could have influenced adherence and clinical outcomes, could not be included. Their exclusion was due to the difficulty in obtaining such information accurately and consistently in a retrospective review. We believe that the findings can be extrapolated to other rural and multicultural regions of Latin America.

## Conclusion

Structural barriers such as road inaccessibility, low educational attainment and belonging to indigenous peoples were significantly associated with lower *r* adherence to radiotherapy and worse clinical outcomes. Therapeutic adherence emerged as a key protective factor for survival. These findings highlight the need to implement specific interventions, such as:

- Hypofractionated regimens, which reduce treatment duration and facilitate adherence.
- Free transport and accommodation programmes for rural patients.
- Intercultural health education, adapted to indigenous languages and worldviews.
- Participation of indigenous community promoters in therapeutic follow-up.
- Training of health personnel in intercultural communication and psychosocial support.

A comprehensive approach to these strategies could substantially improve equity in oncology in Peru and other countries with similar structural inequalities.

## Conflicts of interest

The authors declare that they have no conflicts of interest.

## Funding

The Regional Institute of Neoplastic Diseases funded the data processing.

## Figures and Tables

**Table 1.** Characteristics of the sample treated with radiotherapy from 2020 to 2021.

Factor		*N*	%
Gender	Female	217	85.40
	Male	37	14.60
Indigenous origin	Yes	20	7.90
	No	234	92.10
Productive activity	Agriculture	80	31.50
	Trade	102	40.20
	Manufacturing	28	11.00
	Construction	19	7.50
	Textiles	14	5.50
	Not specified	11	4.30
Residence in provincial capital	No	76	29.90
	Yes	178	70.10
Paved road access	No	115	45.30
	Yes	139	54.70
Education with completed secondary education	No	157	61.80
	Yes	97	38.20
Age	<39 years old	27	10.60
	40–49 years old	61	24.00
	50–59 years old	66	26
	60–69 years old	43	16.90
	70–79 years old	39	15.40
	>80 years old	18	7.10
Tumour site	Stomach and oesophagus	4	1.60
	Breast	27	10.60
	Cervix	131	51.60
	Head and neck	20	7.90
	Skin	9	3.50
	Prostate	5	2.00
	Other	58	22.80
Clinical stage	I	9	3.50
	II	68	26.80
	III	132	52.00
	IV	36	14.20
	Progression	9	3.50
Concurrent chemotherapy	No	94	37.00
	Yes	160	63.00
Adherent	No	111	43.70
	Yes	143	56.30
Treatment interruption	LINAC inoperative	5	2.00
	Personal reasons	51	20.1
	Logistical reasons for relocation	16	6.3
	Toxicity	5	2.0
	Holidays	28	11.00
	Combined	67	26.40
	No interruption	82	32.30

**Table 2.** Factors for non-adherence (multivariate model).

Factor	Adjusted OR	95% CI	*p*-value
Indigenous ethnicity (yes)	10.45	2.34–46.67	0.002
Paved road access (no)	2.19	1.10–4.36	0.026
Incomplete secondary education	2.31	1.33–4.01	0.003

Adjusted OR obtained through multivariate logistic regression. Model adjusted for sex, indigenous status, productive activity, residence, road access, secondary education, age, tumour site, clinical stage and chemotherapy. 95% confidence interval (95% CI).

Statistical significance was considered for *p* < 0.05.

Only significant variables are presented.

**Table 3.** Difference in means for progression-free and overall survival.

Factor		Progression-free survival			Overall survival		
		Mean	SD	*p*-value	Mean	SD	*p*-value
Indigenous affiliation	Yes	6.50	6.82	0.000	10.15	9.65	0.00
	No	31.04	21.99		36.27	19.72	
Productive activity agricultural	Agricultural	19.91	20.84	0.000	25.48	20.10	0.00
	Trade	29.99	22.29		36.37	20.45	
	Manufacturing	38.79	20.18		41.04	18.80	
	Construction	44.47	16.96		47.26	12.84	
	Textiles	19.64	17.19		25.57	15.58	
	Not specified	48.73	8.40		48.91	7.82	
Residence in provincial capital	No	25.41	22.89	0.082	28.89	21.99	0.006
	Yes	30.69	21.77		36.49	19.26	
Paved road access	No	18.49	20.97	0.00	22.13	20.51	0.00
	Yes	37.90	19.18		44.22	13.82	
	70–79 years	27.92	21.88		33.67	21.20	
	≥80 years	20.44	20.92		23.94	18.92	
Tumour site tumour	Stomach and oesophagus	14.25	23.98	0.005	15.50	23.40	0.003
	Breast	43.78	18.38		47.37	14.51	
	Cervix	29.22	22.01		34.81	19.90	
	Head and neck	23.30	19.60		28.50	18.70	
	Skin	34.00	22.09		35.11	21.12	
	Prostate	22.00	24.85		22.80	24.28	
	Other	24.91	22.42		30.86	21.13	
Clinical stage clinical	I	41.44	20.32	0.000	42.22	18.90	0.00
	II	44.94	16.44		46.62	13.72	
	III	25.17	21.38		31.87	20.22	
	IV	15.69	18.85		21.03	20.52	
	Progression	8.56	4.45		19.67	12.60	
Adherence	No	24.49	21.48	0.003	30.71	20.90	0.015
	Yes	32.70	22.16		36.94	19.60	
Interruption	LINAC inoperative	23.60	25.85	0.012	31.80	21.66	0.004
	Personal reasons	22.31	20.97		27.20	20.85	
	Logistical reasons for relocation	27.44	25.28		29.00	24.17	
	Toxicity	7.20	9.47		11.00	10.03	
	Holiday	32.61	21.21		39.29	17.77	
	Combined	28.43	21.80		35.91	19.67	
	No interruption	34.70	21.84		38.05	19.37	

Student's *t*-test was applied for dichotomous variables and ANOVA for polytomous variables

Statistical significance was considered for *p* < 0.05

**Table 4.** Progression-free survival and overall survival according to factors (Kaplan-Meier).

Factor		Progression-free survival			Overall survival		
		Estimate	95% CI	p-value	Estimate	95% CI	*p*-value
Interruption	LINAC inoperative	24.20	3.31 - 45.09	0.001	32.40	14.90 - 49.90	0.002
	Personal reasons	25.88	18.88 - 32.89		30.97	24.16 - 37.78	
	Logistical reasons for relocation	28.44	15.90 - 40.98		30.44	17.55 - 43.32	
	Toxicity	7.20	0.00 - 15.50		13.00	2.08 - 23.92	
	Holiday	34.89	26.08 - 43.70		44.25	36.59 - 51.91	
	Combined	31.74	25.65 - 37.83		42.40	36.78 - 48.02	
	No interruption	39.69	34.13 - 45.69		43.12	38.17 - 48.06	
Adherence	No	26.59	22.12 - 31.05	0.001	35.56	30.99 - 40.13	0.054
	Yes	37.16	32.88 - 41.43		41.64	37.83 - 45.45	
Tarmac road access	No	19.19	15.15 - 23.24	0.00	23.65	19.59 - 27.71	0
	Yes	43.04	39.21 - 46.87		50.76	47.97 - 53.56	

Estimates were made using Kaplan-Meier curves and log-rank test. 95% confidence interval (95% CI).

Statistical significance was considered for *p* < 0.05.

**Table 5.** Implications for clinical practice.

Implication	Feasibility in similar contexts
Implement free transport and accommodation for rural patients.	Requires coordination between ministries of health, local governments and social programmes.
Incorporate indigenous community promoters into support and follow-up.	Can be integrated into existing intercultural health programmes in several Andean and Amazonian countries.
Offer hypofractionated regimens based on available evidence.	Available in reference cancer centres in Latin American countries; scalable with technical training.
Strengthen intercultural health education programmes.	Possible through adaptation of educational material to indigenous languages and dissemination in community media.
Train health personnel in communication and cultural barriers.	Feasible through continuing education modules; incorporated into intercultural health policies.

Summary of proposals derived from the results and their feasibility in similar contexts.
